# MRI of the upper airways in children and young adults: the MUSIC study

**DOI:** 10.1136/thoraxjnl-2020-214921

**Published:** 2020-10-29

**Authors:** Bernadette Elders, Pierluigi Ciet, Harm Tiddens, Wytse van den Bosch, Piotr Wielopolski, Bas Pullens

**Affiliations:** 1 Department of Pediatric Pulmonology, Erasmus MC Sophia, Rotterdam, The Netherlands; 2 Department of Radiology and Nuclear Medicine, Erasmus MC, Rotterdam, The Netherlands; 3 Department of Pediatric Othorhinolaryngology, Erasmus MC Sophia, Rotterdam, The Netherlands

**Keywords:** imaging/CT MRI etc

## Abstract

**Rationale:**

Paediatric laryngotracheal stenosis (LTS) is often successfully corrected with open airway surgery. However, respiratory and vocal sequelae frequently remain. Clinical care and surgical interventions could be improved with better understanding of these sequelae.

**Objective:**

The objective of this cross-sectional study was to develop an upper airway MRI protocol to obtain information on anatomical and functional sequelae post-LTS repair.

**Methods:**

Forty-eight patients (age 14.4 (range 7.5–30.7) years) and 11 healthy volunteers (15.9 (8.2–28.8) years) were included. Spirometry and static and dynamic upper airway MRI (3.0 T, 30 min protocol) were conducted. Analysis included assessment of postoperative anatomy and airway lumen measurements during static and dynamic (inspiration and phonation) acquisitions.

**Main results:**

Good image quality without artefacts was achieved for static and dynamic images in the majority of MRIs. MRI showed vocal cord thickening in 80.9% of patients and compared with volunteers, a significant decrease in vocal cord lumen area (22.0 (IQR 17.7–30.3) mm^2^ vs 35.1 (21.2–54.7) mm^2^, p=0.03) but not cricoid lumen area (62.3±27.0 mm^2^ vs 66.2±34.8 mm^2^, p=0.70). Furthermore, 53.2% of patients had an A-frame deformation at site of previous tracheal cannula, showing lumen collapse during inspiration. Dynamic imaging showed incomplete vocal cord abduction during inspiration in 42.6% and incomplete adduction during phonation in 61.7% of patients.

**Conclusions:**

Static and dynamic MRI is an excellent modality to non-invasively image anatomy, tissue characteristics and vocal cord dynamics of the upper airways. MRI-derived knowledge on postsurgical LTS sequelae might be used to improve surgery.

**Key message**

**What is the key question?**

- Can MRI be used to image the upper airways in patients after laryngotracheal stenosis repair?

**What is the bottom line?**

- Static and dynamic MRI is an excellent modality to non-invasively image anatomy, tissue characteristics and vocal cord dynamics of the upper airways.

**Why read on?**

- This study reports novel findings on the anatomy, tissue characteristics and dynamics of the upper airways after laryngotracheal stenosis repair on MRI.

## Introduction

Paediatric laryngotracheal stenosis (LTS), mostly caused by prolonged intubation, can have lifelong consequences.^1^ Open airway surgery achieves decannulation in up to 95% of severe cases, but respiratory and vocal sequelae may occur.^1–4^ Up to 70% of children have impaired in and expiratory pulmonary function and the majority reports poor voice quality.^5–7^

The gold standard to assess the upper airways is direct laryngotracheoscopy under general anaesthesia.^2 8^ However, this method has important limitations, such as need for sedation, the semiquantitative character of information obtained with possible substantial interobserver variability, suboptimal tissue characterisation and dynamic evaluation, which can be limited by the depth of anaesthesia or the presence of the endoscope in the airway.^9^ To broaden our knowledge on postoperative sequelae, we need a safe and comprehensive imaging method to visualise the upper airways in static and dynamic conditions. CT has been used to image paediatric laryngeal stenosis,^10 11^ but has the important downside of ionising radiation, especially in the region of the radiation-sensitive thyroid.^12^ Thanks to improvements in MRI technology, high-resolution images are achievable in paediatric patients and can be used to evaluate the upper airways.^13^ Notably, the absence of ionising radiation, the improved tissue characterisation and the possibility of dynamic imaging make MRI suitable to image the upper airways after surgery. However, several challenges remain, such as image degradation from motion artefacts and long scan times. Consequently, to date MRI has only been used to detect and grade LTS in adult patients.^14 15^

The aim of this cross-sectional study was to develop a static and dynamic upper airways MRI protocol to obtain information on anatomical and functional sequelae after open airway surgery for paediatric LTS.

## Methods

We invited all patients with a history of open airway surgery for LTS to participate in the **M**RI of the **u**pper airway**s i**n **c**hildren and young adults (**MUSIC**) study. Inclusion and exclusion criteria are shown in online supplemental file 1. Patients were compared with healthy volunteers, consisting of patients’ siblings or friends, without airway, vocal or pulmonary comorbidities. A comparison between patients who did and did not participate in the MUSIC study is shown in online supplemental file 2. Participating patients appeared to be significantly younger than those who did not participate (14.4 (IQR 11.7–19.4) vs 19.1 (14.3–24.0) years, p=0.01). Written informed consent was obtained from all study participants.

10.1136/thoraxjnl-2020-214921.supp1 Supplementary data

10.1136/thoraxjnl-2020-214921.supp2 Supplementary data

### Clinical status

The following data were collected from patients’ electronic medical file: acquired or congenital stenosis, Cotton-Myer grade of stenosis,^16^ location of stenosis (posterior glottis, subglottis, both), presence of a tracheal cannula before repair (Yes/No), type of surgery (single-stage (ss), double-stage laryngotracheal reconstruction (ds-LTR), cricotracheal reconstruction (CTR)),^2^ age at reconstruction and presence of comorbidities. Flow volume spirometry was obtained according to European Respiratory Society/American Thoracic Society guidelines by a certified researcher (BE).^17^ Data are reported as percentage predicted or z-scores according to Gli index.^18 19^

### MRI

**Figure 1:**
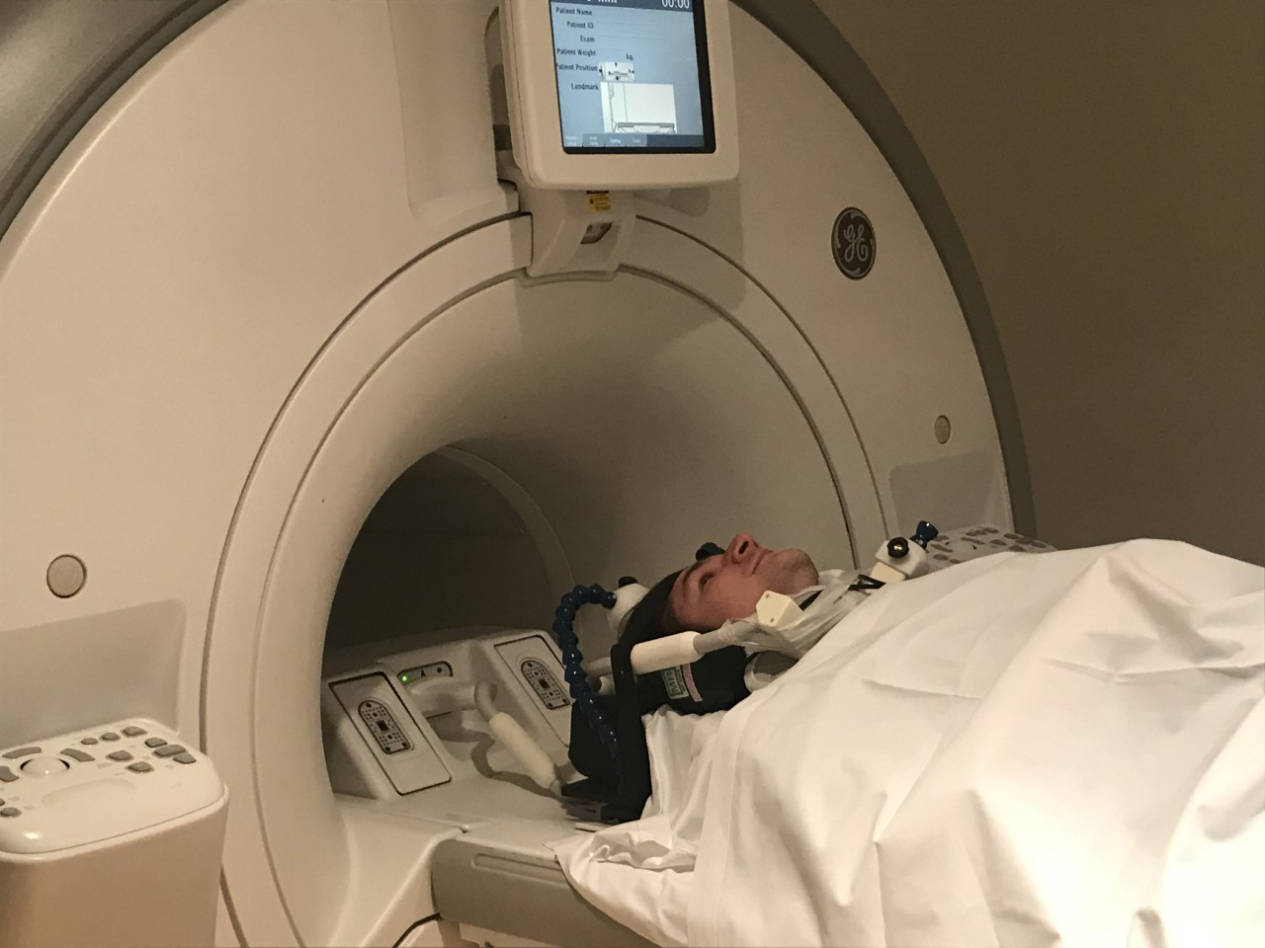
Coil setup, healthy volunteer demonstrating positioning of the 6CH carotid coil placed at the anterior side of the neck and secured with tape, the head is immobilised by the coil placement.

**Figure 2:**
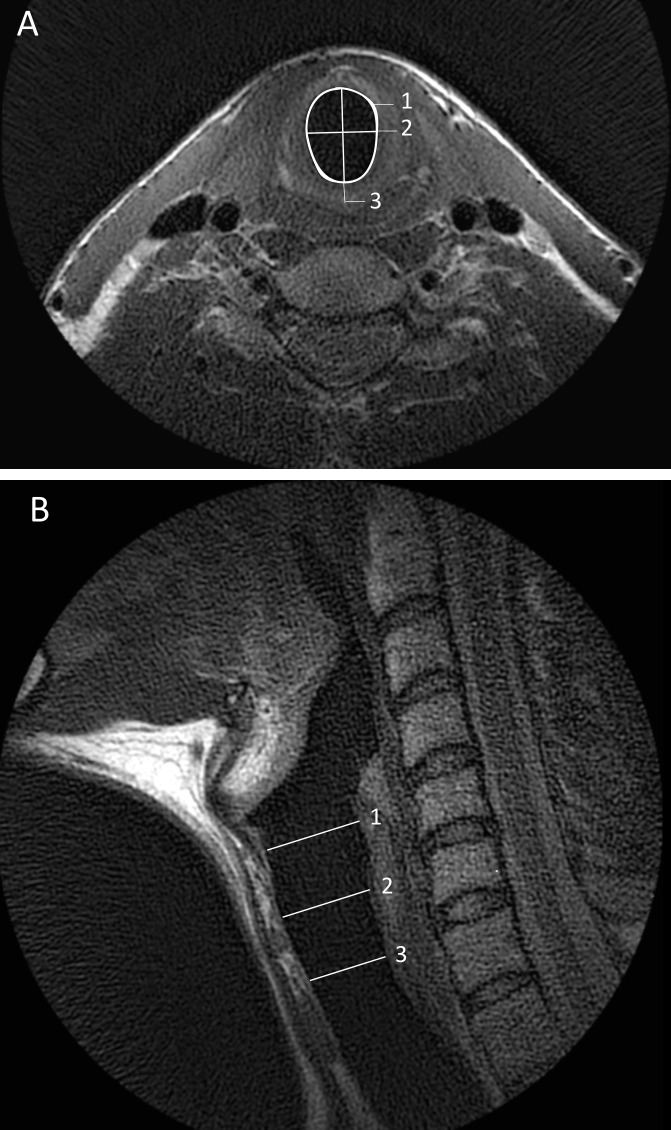
Example of MRI analysis on a healthy volunteer (A) axial T2-weighted image showing the measurement of the lumen area (1), Left right (2) and transversal diameter (3) at the level of the Cricoid, (B) sagittal T2-weighted image showing the location of the cricoid (1), the proximal trachea (2) and the most caudal slice (3).

A paediatric upper airway MRI protocol was developed on a 3.0 T scanner (Discovery MR750, GE Healthcare, Milwaukee, Wisconsin, USA) using a six channel dedicated carotid coil (Machnet B.V., Rhoden, The Netherlands and Flick Engineering B.V., Winterswijk, The Netherlands) (figure 1).^20^ Total protocol duration was 30 min. The protocol consisted of static-free breathing sequences to assess anatomy and tissue characterisation and dynamic sequences to assess upper airway movement. Dynamic sequences consisted of two-dimensional (2D) and 3D sequences, during which the following trained manoeuvres were performed: a manoeuvre of inspiration and ‘AAA’ phonation of 7 s each performed separately during two static 2D sequences and a manoeuvre of 2 s inspiration, followed by 6 s ‘AAA’ phonation during the dynamic 3D sequence. These sequences were repeated until one of the manoeuvres was done correctly. Scan parameters are included in online supplemental file 3A. The principal investigator (PI) (BE) trained participants the manoeuvres and was present during all MRI sessions. Image quality and presence and type of artefacts were recorded. Image analysis was done using Advantage Windows Server platform (V.2.0, GE Healthcare, Milwaukee, Wisconsin, USA), as shown in figure 2, details are provided in online supplemental file 3B.

10.1136/thoraxjnl-2020-214921.supp3 Supplementary data

10.1136/thoraxjnl-2020-214921.supp4 Supplementary data

We assessed anatomy on the T2 (T2-w) and Proton Density-weighted (PD-w) static scans and recorded the presence of arytenoid prolapse or vocal cord thickening, altered vocal cord positioning, presence and displacement of autologous cartilage grafts (anterior, posterior, none) and airway deformations. Airway lumen area, anterior–posterior and transversal diameters were measured on axial T2-w sequences at the level of the vocal cords, cricoid, tracheal deformation (if present) and proximal trachea and corrected for height in metres. Measurement of the proximal trachea was done at the axial slice midway between the cricoid and the most caudal slice. If this was the site of the tracheal deformation, a superior axial slice was used.

We assessed tissue characteristics on T2-w and diffusion-weighted imaging (DWI) to identify structural tissue alterations such as fibrosis, (chronic) oedema or a combination, at the following locations: arytenoids, vocal cords or cricoid.^21 22^ Tissue was characterised as described in table 1.

**Table 1:** Diffusion-weighted imaging (DWI) analysis methods

T2-w imaging		DWI	ADC	Tissue characterisation
Hyper intensity	Thickening			
+	+	Hyper	Hyper	Oedema and fibrosis
+	–	Hyper	Hyper	Oedema
–	+	Hyper	Hyper	Fibrosis
+	+	No signal	No Signal	Mild oedema and fibrosis
+	–	No signal	No signal	Mild oedema
–	+	No signal	No signal	Mild fibrosis
+/–	+/–	Hyper	Hypo	Unknown
+/–	+/–	Hypo	Hyper	Unknown
–	–	Hyper	Hyper	Artefact
–	–	No signal	No signal	Normal tissue

+/–, either present of absent; +, present; –, absent; ADC, apparent diffusion coefficient.

Movement of the upper airways was assessed on 2D and 3D dynamic imaging. Correct performance of the manoeuvres was evaluated in video format, defined as any vocal cord abduction during inspiration and any vocal cord adduction during phonation on 2D images, and any vocal cord movement on 3D images. Movement was evaluated on completeness and symmetry. Airway lumen areas were measured on axial 2D sequences at the vocal cords, cricoid, tracheal deformation (if present) and proximal trachea during inspiration and phonation. Quantitative measurements were only made if 2D manoeuvres were performed correctly.

Patients with a history of CTR, which is characterised by a partially absent cricoid cartilage, were excluded from measurements at the cricoid level. Analysis was done by the PI (BE) with 2 years experience in upper airways MRI. Intraobserver and interobserver reproducibility was tested by reanalysing 12 randomly selected MRIs by the PI and a second researcher appropriately trained in upper airways MRI (WvdB).

### Statistics

The Shapiro-Wilk test was conducted to test data distribution. Normally distributed data are presented as mean±SD and non-normally distributed data are presented as median (range or IQR). Data were compared using the parametric t-test for normally distributed data, the Mann-Whitney U test for not normally distributed data and the χ^2^ test for categorical data. Similarly, Pearson’s and Spearman’s r correlations were calculated for normally and not normally distributed data respectively. Binary logistic regression was used for categorical data. Intraobserver and interobserver agreement was calculated with the intraclass correlation coefficient. Correction for multiple testing was not performed and a 5% significance level was assumed. Data analysis was done using SPSS Statistics (V.25, IBM).

## Results

Patient and healthy volunteer characteristics are presented in table 2. Forty-eight patients and eleven healthy volunteers were included, with a mean age of 14.4 (range 7.5–30.7) and 15.9 (range 8.2–28.8) years. Three patients (6.3%) had a congenital LTS, and 45 patients (93.8%) had an acquired LTS. Patients most often underwent ss-LTR (87.5%). The most common comorbidity was bronchopulmonary dysplasia (BPD) (18.8%).

**Table 2:** Patient and healthy volunteers characteristics

	Patients (n=48)	Volunteers (n=11)	P value
**Age at MRI (years**)	14.4 (range 7.5–30.7)	15.9 (range 8.2–28.8)	0.92
**Gender (n/% female**)	25/52.1	4/36.4	0.37
**Weight (kg**)	45.2 (IQR 34.5–62.3)	60.8 (IQR 31.6–74.0)	0.34
**Height (metre**)	1.6 (IQR 1.4–1.7)	1.7 (IQR 1.4–1.8)	0.34
**Type of stenosis (n/%**)			
Congenital	3/6.3		
Acquired	45/93.8		
**Cotton Myer grade of stenosis (n/%**)			
Grade I	4/8.3		
Grade II	15/31.3		
Grade III	27/56.3		
Grade IV	2/4.2		
**Location of stenosis (n/%**)			
Posterior glottis	11/22.9		
Subglottis	17/35.4		
Posterior glottis and subglottis	20/41.7		
**Tracheal cannula before repair (n/%**)	38/79.2		
**Type of reconstruction (n/%**)			
ss-LTR	42/87.5		
ds-LTR	2/4.2		
CTR	4/8.3		
**Age at reconstruction (years**)	2.2 (IQR 1.1–4.5)		
**Years since reconstruction**	11.5±4.6		
**Comorbidities (n/%**)	Asthma 2/4.2		
	BPD 9/18.8		
	Cardiac 3/6.3		
	DiGeorge syndrome 1/2.1		
	Oesophageal atresia 3/6.3		
	Kartagener syndrome 1/2.1		
	OSAS 1/2.1		
	PMR 6/12.5		
	Tracheomalacia 2/4.2		

Descriptive statistics. Data are presented as mean±SD, median (range or IQR) or absolute number (n) and percentage.

BPD, bronchopulmonary dysplasia; CTR, cricotracheal resection; ds-LTR, double stage laryngotracheal reconstruction; OSAS, obstructive sleep apnoea syndrome; PMR, psychomotor retardation; ss-LTR, single-stage laryngotracheal reconstruction.

Table 3 shows spirometry data for patients and healthy volunteers. Patients’ expiratory and inspiratory (expressed as a decrease in forced inspiratory volume in 1 s (FIV_1_) divided by maximal vital capacity (VCmax) and an increase in Expiratory Disproportion Index (EDI)) spirometry outcomes were lower than those of healthy volunteers. Spirometry outcomes did not differ significantly between LTS patients with and without BPD (forced expiratory volume in 1 s (FEV_1_) z-score=−1.9 ± 1.7 vs -0.2±1.00, p=0.88, FEV_1_/functional vital capacity (FVC) z-score=−2.3 (IQR -2.9 to −0.7) vs −1.2 (−2.0 to −0.3), p=0.34, FIV_1_/VCmax=65.6 (IQR 49.8–80.0) vs 63.2 (54.2–80.1), p=0.84, EDI=67.6% ± 15.0% vs 64.6±15.4%, p=0.94).

**Table 3:** Flow volume spirometry data

	Patients (n=48)	Volunteers (n=11)	P value
**FVC**			
% predicted	86.5 (80.5–100.0)	97.0 (91.0–110.0)	0.02*
z-score	−1.0±1.3	0.1±1.1	0.02*
**FEV_1_**			
% predicted	80.5 (73.0–91.0)	97.0 (91.0–107.0)	0.001*
z-score	−1.6 (−2.3 to −0.8)	−0.3 (−0.8 to 0.6)	0.001*
**FEV_1_/FVC**			
% predicted	91.0 (79.3–96.0)	100 (94.0–106.0)	0.02*
z-score	−1.4±1.6	−0.3±1.6	0.04*
**PEF**			
% predicted	58.5 (41.3–71.8)	111.0 (78.0–123.0)	<0.001*
z-score	−3.1 (−4.4 to −2.2)	0.6 (−1.6 to 1.6)	<0.001*
**FEF25**			
% predicted	61.5 (43.5–61.5)	109.0 (76.0–127.0)	<0.001*
z-score	−2.8±1.4	−0.3±2.2	<0.001*
**FEF50**			
% predicted	62.1±21.8	93.1±44.8	<0.001*
z-score	−2.4±1.5	−0.8±2.3	0.007*
**FEF75**			
% predicted	81.5±36.2	104.4±44.8	0.08
z-score	−0.8±1.4	−0.1±1.4	0.11
**MEF**			
% predicted	64.0±23.0	92.8±31.7	0.001*
z-score	−1.8±1.3	−0.4±1.5	0.003*
**VCmax**			
% predicted	89.1±15.2	100.7±12.7	0.02*
z-score	−1.1 (−1.53–0.0)	−0.3 (−0.8–0.9)	0.02*
**FVC in**			
% predicted	75.5±15.0	85.7±15.4	0.05*
**FIV_1_ (L**)	1.7 (1.3–2.5)	2.7 (1.6–5.6)	0.47
**FIV_1_/FVC in (%**)	78.5 (65.0–92.1)	99.8 (88.2–100.0)	0.01*
**FIV_1_/VCmax (%**)	66.3±16.5	80.9±9.5	0.007*
**FEV_1_/FIV_1_ (%**)	119.0±25.8	105.3±17.6	0.1
**EDI (%**)	62.6 (52.5–79.4)	45.3 (43.9–50.1)	0.001*

Spirometry data. Data are presented as mean±SD or median (IQR).

*P<0.05.

EDI, Expiratory Disproportion Index; FEF_25-75_, forced expiratory flow at 25%–75% of expiration; FEV_1_, forced expiratory volume in 1 s; FIV_1_, forced inspiratory volume in 1 s; FIV in, forced inspiratory volume; FVC, forced vital capacity; FVC in, forced vital capacity inspiratory; MEF, mean expiratory flow; PEF, peak expiratory flow; VC max, maximum vital capacity.

### MRI

MRI was successfully performed in 47 patients and 10 healthy volunteers (97%) (table 4). Two participants did not complete the MRI protocol due to claustrophobia. Image quality of the majority of the images was good, without artefacts in 66.7%. Excellent image resolution was achieved with best spatial resolution of 0.5×0.5 (in plane) x 2 mm (slice thickness) and best temporal resolution of 330 ms (3D dynamic scans).

**Table 4:** MRI results

General quality	Patients and volunteers=59		
**Successful MRI (n/%**)	57/97		
**Quality static images (n/%**)	Bad 0/0.0		
	Fair 2/3.5		
	Moderate 8/14.0		
	Good 35/61.4		
	Excellent 12/21.1		
**Quality dynamic images (n/%**)	Bad 2/3.5		
	Fair 5/8.8		
	Moderate 6/10.5		
	Good 40/70.2		
	Excellent 4/7.0		
**Artefacts (n/%**)	None 38/66.7		
	Coil 12/21.1		
	General motion 6/10.5		
	Respiratory motion 1/1.8		
**Static**	**Patients=47†**	**Volunteers=10**	**P value**
**Vocal cord thickening (n/%**)	38/80.9	1/10	
**Abnormal vocal cord positioning (n/%**)	10/21.3	1/10	
**Vocal cords**			
Area (mm^2^)	22.0 (17.7–30.3)	35.1 (21.2–54.7)	0.03*
AP diameter (mm)	8.4±2.0	9.0±2.3	0.37
Transversal diameter (mm)	2.8±1.0	3.8±1.2	0.01*
**Cricoid**†			
Area (mm^2^)	62.3±27.0	66.2±34.8	0.70
AP diameter (mm)	8.4±2.0	9.8±2.1	0.05
Transversal diameter (mm)	6.3±1.4	5.7±0.8	0.16
**Presence of tracheal deformation (n/%**)	25/53.2	0/0	
**Tracheal deformation**			
Area (mm^2^)	28.6 (20.8–41.9)	–	
AP diameter (mm)	6.6±1.7	–	
Transversal diameter (mm)	4.4±1.1	–	
**Proximal trachea**			
Area (mm^2^)	61.0 (39.9–83.3)	86.1 (41.5–130.1)	0.19
AP diameter (mm)	7.3±1.5	8.1±2.1	0.16
Transversal diameter (mm)	7.3±1.6	8.2±1.3	0.10
**Area decrease at deformation (area deformation/area trachea,%**)	37.5±23.5	–	–
**Dynamic**			
**Complete abduction during inspiration (n/%**)	27/57.4	10/100	0.01*
**Complete adduction during phonation (n/%**)	18/38.3	10/100	<0.01*
**2D**	**Patients=35†**	**Volunteers=9**	
**Inspiration areas (mm^2^**)			
Vocal cords	43.7±22.7	72.9±42.3	0.01*
Cricoid	67.5±26.9	84.3±42.4	0.15
Tracheal deformation	38.4±13.7	–	–
Trachea	79.5±28.5	104.2±50.5	0.07
**Phonation areas (mm^2^**)			
Vocal cords	10.9±9.3	7.2±4.7	0.26
Cricoid	65.6±30.4	90.5±44.4	0.06
Tracheal deformation	46.9±16.7	–	–
Trachea	77.0±27.7	105.2±49.9	0.03*
**Difference in areas (%**)			
Vocal cords	−72.1±23.7	−88.9±7.3	0.04*
Cricoid	−7.5 (−22.7–6.0)	−5.5 (−0.4–19.0)	0.02*
Tracheal deformation	27.0±41.8	–	–
Trachea	−3.4 (−10.4–8.0)	2.2 (−4.6–10.5)	0.21

Data are presented as mean±SD, median (IQR) or absolute numbers (n) and percentages. All areas and diameters are corrected for height in metres.

*P<0.05, data are corrected for height in metres.

†Cricoid measurements conducted in n=43 for static and n=33 for dynamic images, due to exclusion of patients with history of CTR.

AP, anterior–posterior; CTR, cricotracheal reconstruction; 2D, two dimensional.

**Figure 3:**
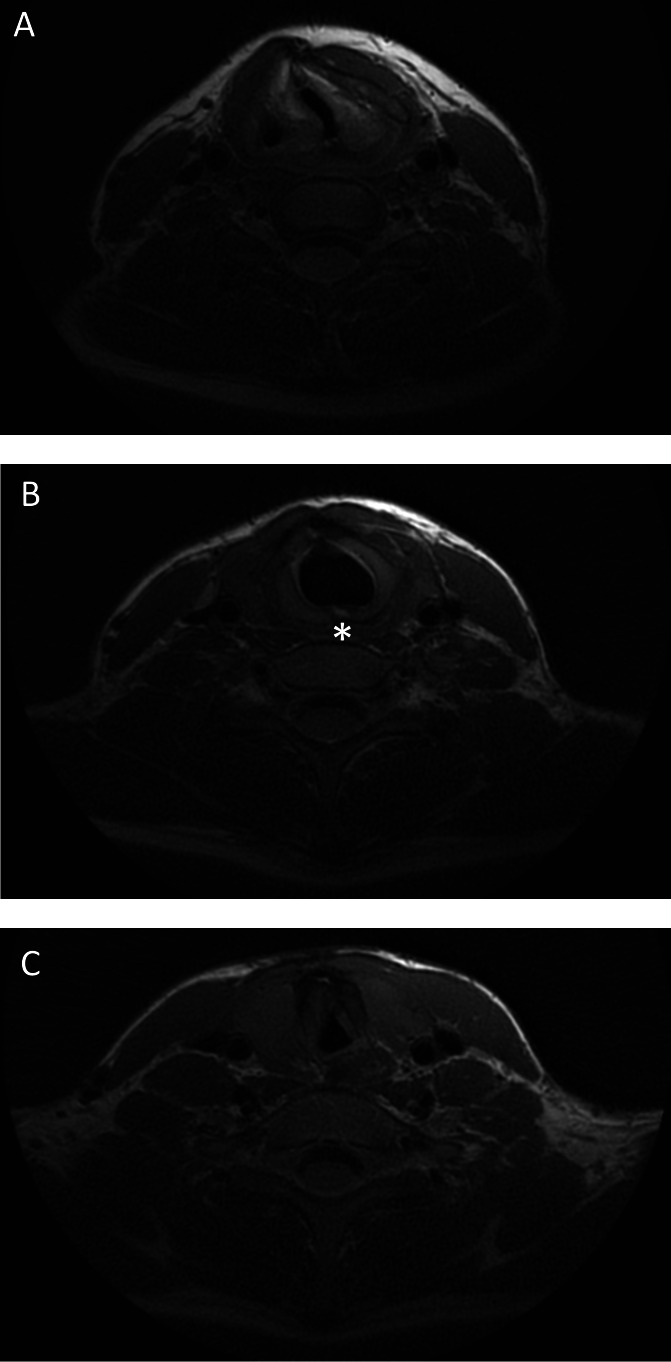
Example of static MRI image of a patient with a history of LTS after prolonged intubation due to prematurity having undergone both an SS-LTR and ds-LTR, showing deviation and thickening of the vocal cords (A), asterisk indicating the posterior cartilage graft placed during LTR (B) and the A-frame deformation of the trachea (C). ds-LTR, double stage laryngotracheal reconstruction; LTS, laryngotracheal stenosis; SS, single stage.

Figure 3 shows an example of a patient’s static MRI. The outcomes of the static and dynamic MR analyses are shown in table 4. Thirty-eight patients (80.9%) and one healthy volunteer (10%) showed vocal cord thickening on MRI. This volunteer had a history of Tetralogy of Fallot, and had repeatedly been intubated during surgery, without any current complaints of respiratory distress. In patients with a history of LTR (n=43) autologous cartilage grafts could be identified on MRI in all but one patient, in whom the anterior graft could not be seen due to an artefact caused by a recently repaired tracheal fistula. Thirteen patients (30.2%) showed displacement of cartilage graft(s) into the airway lumen, namely displacement of the anterior cartilage graft in 3/39 (7.7%) and displacement of the posterior cartilage graft in 12/41 (29.3%) patients. Online supplemental file 4 shows an example of displacement of a posterior cartilage graft.

10.1136/thoraxjnl-2020-214921.supp5 Supplementary data

Patients’ vocal cord lumen area was significantly smaller than that of healthy volunteers during free breathing. At the cricoid level, the location of LTR, the lumen area was comparable between patients and healthy volunteers. Additionally, in 25 patients (53.2%) an A-frame deformation at the level of previous tracheal cannula was found with an average lumen area reduction of 37.5% (range −9.3% to −74.5%) compared with the proximal trachea. Three patients showed other trachea deformations: one idiopathic saber-sheath deformation (an intrathoracic tracheal deformation in which the transversal diameter of the trachea is smaller than the anterior–posterior diameter),^23^ one saber-sheath deformation with an oesophageal pouch after oesophageal atresia repair, and one known malacia. In 78.3% of patients the narrowest point of the airway was located at the vocal cords, in 19.6% at the tracheal deformation, and in 2.2% at the trachea. In all healthy volunteers the narrowest point was located at the vocal cords.

A significant correlation between vocal cord lumen area and both in- and expiratory spirometry was found (table 5, online supplemental file 5).

10.1136/thoraxjnl-2020-214921.supp6 Supplementary data

**Table 5:** Correlations between areas at different levels of the upper airways on MRI and spirometry

	FEV_1_ (%pred)		FEV_1_/FVC (%pred)		FIV_1_/VCmax (%)		EDI (%)	
Vocal cord area	r_s_=0.30 (0.04 to 0.52)	**p=0.02**	r_s_=0.33 (0.06 to 0.54)	**p=0.01**	r=0.34 (0.08 to 0.58)	**p=0.01**	r_s_=−0.31 (−0.53 to 0.05)	**p=0.02**
Cricoid area	r=0.03 (−0.24 to 0.29)	p=0.85	r=−0.02 (−0.29 to 0.25)	p=0.88	r=0.03 (−0.24 to 0.29)	p=0.84	r=−0.08 (−0.19 to 0.35)	p=0.54
Tracheal deformation area	r_s_=0.25 (−0.16 to 0.59)	p=0.22	r_s_=0.19 (−0.23 to 0.54)	p=0.37	r=0.22 (−0.20 to 0.63)	p=0.30	r_s_=−0.01 (−0.19 to 0.35)	p=0.97
Trachea area	r_s_=0.18 (−0.09 to 0.42)	p=0.19	r_s_=0.14 (−0.13 to 0.39)	p=0.30	r=0.28 (0.01 to 0.53)	**p=0.04**	r_s_=−0.06 (−0.32 to 0.20)	p=0.65

Correlations between areas at different levels of the upper airways on MRI and spirometry. Data are presented as Pearson (r) or Spearman (r_s_) correlation coefficient (95% CI) and p value. P values <0.05 are in bold.

EDI, Expiratory Disproportion Index; FEV_1_, forced expiratory volume in 1 s; FIV_1_, Forced inspiratory volume in 1 s; FVC, forced vital capacity; VCmax, maximal vital capacity.

As an incidental finding MRI showed hypointense regions on PD-w images in 40 patients (85.1%), and none of the healthy volunteers. Ultrasonography in four patients did not clarify the origin of this artefact (online supplemental file 6), being either postsurgical artefacts from the use of metal surgical instruments, such as those detected in gradient-echo imaging of the postsurgical knee,^24 25^ or subcutaneous emphysema, despite on physical examination no crackling could be felt on the neck.

10.1136/thoraxjnl-2020-214921.supp7 Supplementary data

An online supplemental video shows an example of the dynamic MRI of a patient. Complete abduction during inspiration and complete adduction during phonation was seen in 57.4% and 38.3% of patients, respectively, compared with 100% for both in healthy volunteers. In 11 patients (23.4%) asymmetrical vocal cord movement was observed. Quantitative analyses on the 2D dynamic images could be performed in 37 patients (78.7%) and 9 healthy volunteers (90%), due to correct execution of the manoeuvres. In two patients, 2D dynamic scans were of insufficient quality to perform the area measurements. Patients showed a significantly smaller vocal cord lumen area during inspiration compared with healthy volunteers. Binary logistic regression did not show a correlation between posterior glottic involvement of the stenosis and the area between the vocal cords during inspiration (OR 0.98, 95% CI 0.94 to 2.01, p=0.18) nor phonation (OR 1.03, 95% CI 0.95 to 1.13, p=0.46) on MRI. A greater area between the vocal cords during inspiration was correlated to an increase in FIV_1_/VCmax (r=0.38, 95% CI 0.09 to 0.69, p=0.01) but not to the EDI (r=−0.23, 95% CI −0.54–0.07, p=0.13).

10.1136/thoraxjnl-2020-214921.supp8 Supplementary data

In patients with an A-frame trachea deformation, an average decrease in airway lumen area of 27% during inspiration was observed. We did not find a correlation between the presence of a tracheal deformation and a decrease in inspiratory spirometry (FIV_1_/VCmax OR 0.98, 95% CI 0.94 to 1.02, p=0.35 and EDI 1.01, 95% CI 0.96 to 1.05, p=0.77)

On T2-w sequences 37 (78.7%) patients showed signs of fibrosis or chronic oedema, located at the arytenoids in 59.6%, vocal cords in 57.4% and cricoid in 8.5%. Forty- six patients (95.8%) and 10 healthy volunteers (90.9%) had DW images available for analyses. On DWI 26.1% of patients showed signs of oedema, 6.5% of patients showed signs of fibrosis, and 17.4% of patients showed signs of a combination of oedema and fibrosis. One patient had a pattern on hyperintense signal on DWI with hypointense signal on apparent diffusion coefficient (ADC) and one patient had a pattern of hypointense signal on DWI with hyperintense signal on ADC, both of unknown origin. Eighteen patients (39.1%) had signs of tissue alteration on T2-w imaging without signal abnormalities on DWI, corresponding to mild fibrosis and/or oedema. Tissue alteration according to the combination of T2-w and DW imaging did not correlate to previous glottic involvement (1.7, 95% CI 0.5 to 5.9, p=0.40) nor to impaired vocal movement on MRI (0.86, 95% CI 0.3 to 2.7, p=0.79). Tissue alteration according to T2-w images alone, therefore, also including mild fibrosis and oedema, was significantly correlated to previous glottic involvement of the stenosis (OR 9.5, 95% CI 1.7 to 53.4, p=0.01) as well as impaired vocal cord movement on MRI (3.5, 95% CI 1.0 to 11.6, p=0.04).

Intravariability and intervariability analysis showed good to excellent consistency for all static (online supplemental file 7) and dynamic MRI measurements, except for the subjective scoring of complete abduction and adduction.

10.1136/thoraxjnl-2020-214921.supp9 Supplementary data

## Discussion

We used static and dynamic MRI to evaluate the upper airways after open airway surgery for paediatric LTS. MRI enabled visualisation of anatomical structures, tissue characterisation and quantification of vocal cord and airway dynamics and thereby provided a new imaging modality to assess postsurgical sequelae of LTS.

Static MRI permitted quantification of the postsurgical upper airway anatomy in an objective manner. Patients’ vocal cord lumen area, but not cricoid lumen area, was smaller than that of healthy volunteers. These static measurements proved to be highly reproducible as shown by the excellent intraobserver and interobserver agreement. Furthermore, we could visualise the correct or incorrect position of autologous cartilage grafts in the cricoid.

Dynamic MRI of the vocal cords showed a high prevalence of impaired vocal cord movement. These sequences showed to be important to assess respiratory and vocal sequelae of LTS surgery and could be used as monitoring tool for different treatment options.^5^ However, subjective scoring of impaired vocal cord abduction and adduction resulted in poor interobserver agreement, presumably because abduction and adduction are highly influenced by both thickening and abnormal positioning of the vocal cords. Therefore, we did not use subjective scoring of abduction and adduction for further analyses. To evaluate vocal cord movement, we recommend to use area measurements during inspiration and phonation. We furthermore recommend the use of both 2D and 3D sequences, with 2D best suitable for quantitative measurements and 3D best suitable for the overall evaluation of upper airway dynamics.

Interestingly, dynamic MRI showed a high incidence of tracheal deformations with lumen collapse during inspiration. Previous studies have reported a lower incidence of A-frame deformations of 10% after tracheal cannula removal.^26^ Likely the higher incidence in our study related to the ability to detect all A-frame deformations, irrespective of clinical symptoms. We did not observe a correlation between the presence of a tracheal deformation and impaired inspiratory spirometry outcomes. This could either be caused by a small sample size, or more likely by the malacic character of the tracheal deformation, where elastic recoil due to an increased negative pressure at the level of the stenosis (Bernoulli-Venturi effect) varies airway resistance at the level of the stenosis.^27 28^

Tissue characterisation was evaluated on T2-w and DW imaging, showing good correlation between tissue alteration according to T2-w imaging and patient characteristics and outcome. Tissue alteration of airway mucosa (such as the presence of fibrosis) is currently subjectively diagnosed by laryngotracheoscopy, known to be sensitive to observer bias and poor interobserver correlations.^29 30^ T2-w MRI can be used as a replacement as it allows to visualise oedema and fibrosis. In addition, previous studies have shown the potential additional benefit of DWI to detect tissue with decreased free movement of hydrogen molecules due to cellular swelling or increased tissue density, as has been observed in postradiation fibrosis.^13 31 32^ This is the first study using DWI to compare normal airway tissue to postsurgical tissue alterations. Although we did not have histology samples available for comparison, T2-w imaging seems to be able to characterise tissue of the upper airways. No added benefit of DWI compared with T2-w imaging was found, either because DWI does not detect mild tissue alterations or because DWI is highly sensitive to technical variations such as poor signal-to-noise ratios from poor coil fitting, inadequate fat suppression or motion artefacts in younger children. Contrast enhancement might be an alternative method to further improve tissue characterisation, but this would require intravenous access and could raise concerns related to the use of MRI contrast agents as tissue deposition in the brain has been reported.^33^ In selected patients, a combination of MRI with positron emission tomography might be used to detect active inflammation.^34^

Our overall findings suggest that MRI, when performed successfully, can supply highly relevant information for follow-up evaluation of LTS patients and provide similar information as laryngotracheoscopy. MRI can overcome important downsides of laryngotracheoscopy such as the need for anaesthesia, inter-observer variability issues and it allows objective tissue characterisation. A challenge of our MRI protocol is the need for patient cooperation, which precludes its use in children below the age of 6 years and in older non-cooperative children. Good correlations between spirometry outcomes and lumen areas suggest that MRI measurements could aid in understanding the anatomical substrate causing airflow obstruction, as indicated by a previous study.^14^ A more affordable and widely available alternative method could be flexible nasal endoscopy, however, this method is unsuitable to evaluate anatomy below the vocal cords, is non- quantitative and can be challenging in case of arytenoid prolapse. Therefore, we consider flexible nasal endoscopy not a realistic alternative to laryngotracheoscopy nor to MRI.^2^

Our findings also show the diversity and complexity of the postsurgical airway with patients presenting with a heterogeneity in the level(s) and severity of airway obstructions. The effect of obstructions at consecutive airway levels on airway dynamics and airway resistance is not entirely clear. We are currently working on computational fluid dynamic modelling studies to visualise airway mechanics in an attempt to determine the contributions of separate obstructions on airflow patterns and airway resistance.

The main limitation of our study is the lack of direct comparison with the gold standard, laryngotracheoscopy, because this is not routinely performed in our LTS follow-up. However, in four patients, we could compare MRI to a recent laryngotracheoscopy, showing that unilateral vocal cord movement on MRI corresponds to paralysis or asymmetrical fibrosis of the vocal cords on laryngotracheoscopy. This shows that these modalities are comparable, as seen in previous studies in paediatric OSAS and tracheomalacia,^35 36^ as well as between CT and laryngoscopy in tracheomalacia.^37^ Another important limitation to our study is the effect of comorbidities on the lung function test. Comorbidities might have influenced the airflow limitations in our cohort, as spirometry was technically challenging for some of these children. In addition z-scores for inspiratory spirometry values are not available. Lastly, because of the innovative character of our imaging analyses, we could not compare our data to reference values or other studies. We selected healthy volunteers first to get an impression of the healthy upper airways on MRI. For logistic reasons the chosen siblings and friends were not matched for sex and age and no sample size calculation was performed. Therefore, correlations found should be interpreted cautiously and future studies should focus on obtaining MRI reference values at different ages in a larger cohort of healthy children.

In conclusion, this is the first study reporting follow-up MRI data of a large LTS population. MRI proved to be an excellent modality to non-invasively image anatomic structures, characterise tissue and quantify dynamics of the upper airways. Our observations of residual stenosis at consecutive airway levels, a high incidence of A-frame deformations of the trachea, and a high incidence of impaired vocal cord movement, contribute to the knowledge on postsurgical sequelae of LTS. The information provided by MRI might be useful to assess post-LTS sequelae in an out-patient setting. This can be especially helpful in cases of complex multi-level stenosis where imaging can provide the surgeon an objective measurement of the different stenosis sites and can aid in the decision making for further surgery. Our study shows that MRI is a non-invasive safe and valuable image modality for the follow-up of LTS patients.
