# Supplementary material for: MRI of the upper airways in children and young adults: the MUSIC study
Source: Thorax. 2020 Oct 29;76(1):44–52. doi: 10.1136/thoraxjnl-2020-214921 (PMC7803889; doi:10.1136/thoraxjnl-2020-214921)
Supplement: Supplementary data [file thoraxjnl-2020-214921supp002.pdf]

Online supplement 2: Comparison between patients who did and did not participate in the MUSIC study

	**Patients participating=48**	**Patients not participating=35**	
**Age (years)**	14.4 (11.7-19.4)	19.1 (14.3-24.0)	*0.01
**Gender (% female)**	52.1	42.9	0.41
**Type of stenosis (%)**			0.12
Acquired	93.8	82.9	
Congenital	6.3	17.1	
**CM grade of stenosis (%)**			0.08
Grade I	8.3	-	
Grade II	31.3	20.6	
Grade III	56.3	79.4	
Grade IV	4.2	-	
**Location of stenosis (%)**			0.10
Posterior glottis	22.9	17.1	
Subglottis	35.4	57.1	
Posterior glottis and subglottis	41.7	22.9	
**Tracheal cannula before repair (%)**	79.2	74.3	0.60
**Type of repair (%)**			0.95
SS-LTP	87.5	85.7	
DS-LTP	4.2	5.7	
CTR	8.3	8.6	
**Bronchopulmonary dysplasia (%)**	18.8	22.9	0.65

Online supplement 2: Comparison between patients who did and did not participate in the MUSIC study. CM; Cotton Myer, CTR; cricoid resection, ds-LTR; double stage laryngotracheal stenosis, ss-LTR; single stage laryngotracheal reconstruction. Data are presented as median (interquartile range) or percentage. *=p<0.05
