# Supplementary material for: MRI of the upper airways in children and young adults: the MUSIC study
Source: Thorax. 2020 Oct 29;76(1):44–52. doi: 10.1136/thoraxjnl-2020-214921 (PMC7803889; doi:10.1136/thoraxjnl-2020-214921)
Supplement: Supplementary data [file thoraxjnl-2020-214921supp003.pdf]

Online supplement 3A: Upper airways MRI protocol

Sequence	Plane	TR (ms)	TE (ms)	Flip (°)	BW (Hz)	NEX	pif	FOV (cm^2)	Matrix	Slices / thick (mm)	Actual Voxel resolution (mm^3)	Interp. resolution (mm^3)	Time
**Anatomical**													
**2D T2w PROPELLER**	Axial	4400	49	90/ 142	62.5	6	3	15.3 x 15.3	320 x 320	25 / 2.0	0.47 x 0.47 x 2.0	0.29 x 0.29 x 2.0	4 min 46 s
**2D T2w fatsat PROPELLER**	Axial	4570	50	90/ 142	62.5	6	3	15.3 x 15.3	320 x 320	25 / 2.0	0.47 x 0.47 x 2.0	0.29 x 0.29 x 2.0	4 min 57 s
**2D T2w PROPELLER**	Sagittal	3730	50	90/ 142	62.5	6	3	15.3 x 15.3	320 x 320	21 / 2.0	0.47 x 0.47 x 2.0	0.29 x 0.29 x 2.0	4 min 4 s
**2D T2w PROPELLER**	Coronal	3700	50	90/ 142	62.5	6	3	15.3 x 15.3	320 x 320	21 / 2.0	0.47 x 0.47 x 2.0	0.29 x 0.29 x 2.0	4 min 4 s
**3D PDw SPGR Calc**	Axial	3.8	2.1	3	90.9	5	1	15.3 x 15.3	320 x 224	68 / 0.8	0.47 x 0.68 x 0.8	0.29 x 0.29 x 0.4	4 min 3 s
**Tissue Characterization**													
**2D Multi b-value DWI**	Axial	2100	54.6	90/ 180	250.0	3 (b10)<br>5 (b100)<br>7 (b200)<br>11 (b400)<br>15 (b800)	1	18.0 x 4.5	90 x 180	16 / 3.2	2.0 x 1.0 x 3.2	0.70 x 0.70 x 3.2	4 min 17 s
**Dynamic**													
**2D SSFSE** Insp, AAAA	Axial	∞	47	90/ 110	62.5	0.5	2	15.3 x 10.7	160 x 128	25 / 2.0	0.95 x 0.68 x 0.8	0.29 x 0.29 x 0.4	7 s
**3D TRICKS** Cont. Insp-AAAA	Axial	2.0	0.9	3	90.9	1.0	2	21.0 x 12.6	160 x 98	18 / 3.0	1.31 x 2.14 x 3.0	0.82 x 0.82 x 1.5	8 s / 330 ms temporal resolution per volume

Online supplement 2A: Paedatric upper airway MRI protocol. A/P; Anterior/Posterior, BW; Bandwidth, DWI; Diffusion Weighted Imaging, ETL; Echo Train Length, FOV; Field Of View, PD; Proton Density, NEX; Number of Excitations, PROPELLER; Periodically Overlapping ParalLEL Lines with Enhanced Reconstruction , SPGR; Spoiled Gradient Echo Sequence, SSFSE; Single Shot Fast Spin Echo, TE; Echo Time, TR; Repetition Time, TRICKS; Time Resolved Imaging of Contrast Kinetics
