# Supplementary material for: MRI of the upper airways in children and young adults: the MUSIC study
Source: Thorax. 2020 Oct 29;76(1):44–52. doi: 10.1136/thoraxjnl-2020-214921 (PMC7803889; doi:10.1136/thoraxjnl-2020-214921)
Supplement: Supplementary data [file thoraxjnl-2020-214921supp004.pdf]

Online supplement 3B: MRI analysis protocol

Image quality

Bad: major artefacts and blurring, not suitable for analyses
Poor: moderate artefacts and blurring, partially suitable for analyses
Moderate: minor artefacts and blurring, suitable for analyses
Good: no artefacts or blurring, suitable for analyses
Excellent: no artefacts or blurring, excellent delineation of all structures

Artefacts
Coil, general motion or respiration related

Static images

Vocal cord thickening: protrusion of the vocal cords into the airway lumen at the vocal cords
Arytenoid prolapse: protrusion of the arytenoids into the airway lumen at the vocal cords

Vocal cord deviation: deviation from the triangular shape between anterior commissure and vocal cords

Airway deformations: disruption of the circular airway lumen (an A-frame deformation due to previous tracheal cannula placement or other deformations, such as due to esophageal atresia)

Displacement of cartilage grafts: protrusion of the cartilage grafts into the airway lumen

Fibrosis/chronic edema: tissue thickening or hyperintense signal on T2 weighted imaging

Dynamic images

Correct performance of the 2D dynamic maneuvers: any vocal cord abduction during inspiration and any vocal cord adduction during phonation

Correct performance of the 3D dynamic maneuvers: any vocal cord movement

Complete abduction: opening of the vocal cords into a triangular shape with a symmetrical 90 degree angle

Complete adduction: parallel closing of the vocal cords

Inspiration area: area between the vocal cords during inspiration measured on the axial 2D dynamic image

Phonation area: area between the vocal cords during phonation measured on the axial 2D dynamic image

Difference in area: area between the vocal cords during phonation divided by the area between the vocal cords during inspiration x 100%
