# Supplementary material for: MRI of the upper airways in children and young adults: the MUSIC study
Source: Thorax. 2020 Oct 29;76(1):44–52. doi: 10.1136/thoraxjnl-2020-214921 (PMC7803889; doi:10.1136/thoraxjnl-2020-214921)
Supplement: Supplementary data [file thoraxjnl-2020-214921supp005.pdf]

Online supplement 4: Example of posterior cartilage graft displacement

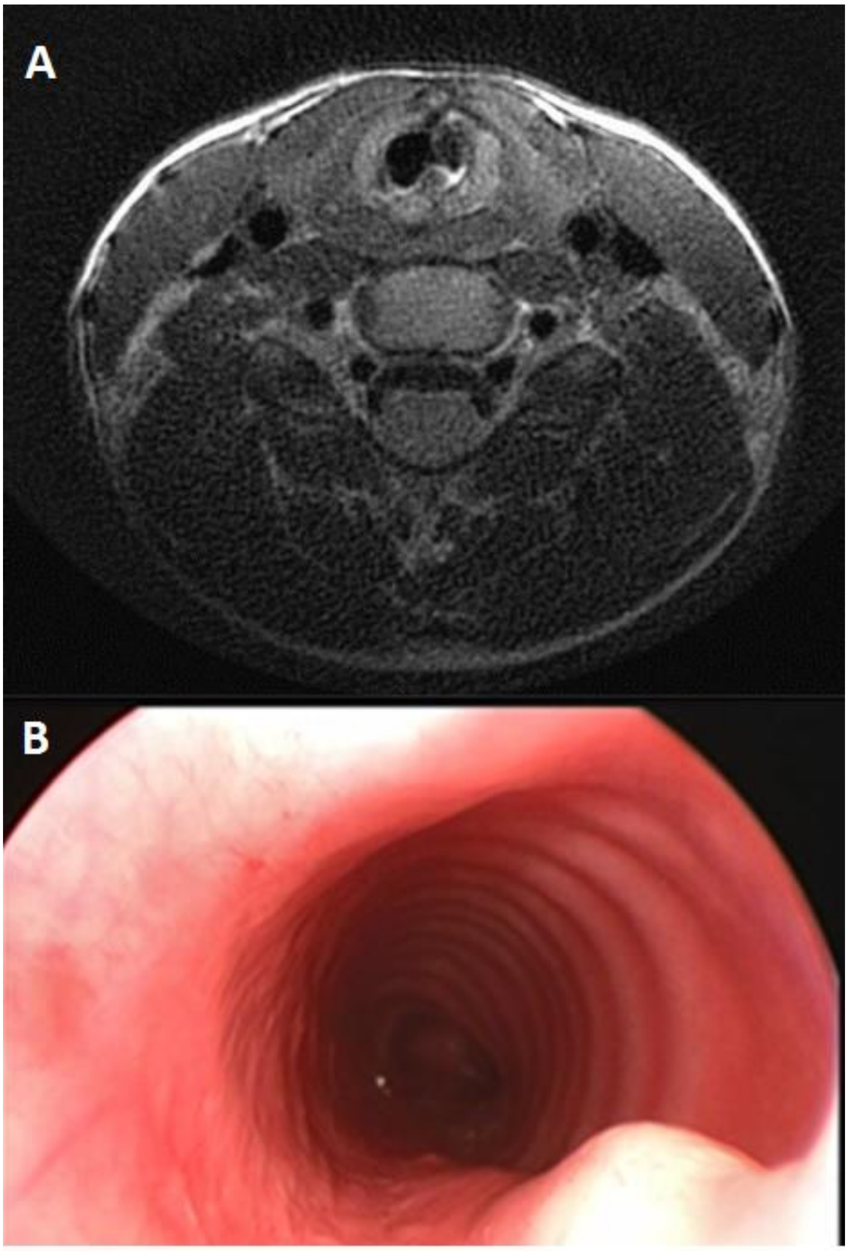

Online supplement 4: example of a patient with history of LTS showing posterior cartilage graft displacement on an axial T2 weighted MR image (A) and laryngoscopy (B). The MR image is mirrored to correspond to the laryngoscope image.
