# Supplementary material for: MRI of the upper airways in children and young adults: the MUSIC study
Source: Thorax. 2020 Oct 29;76(1):44–52. doi: 10.1136/thoraxjnl-2020-214921 (PMC7803889; doi:10.1136/thoraxjnl-2020-214921)
Supplement: Supplementary data [file thoraxjnl-2020-214921supp006.pdf]

**Online supplement 5:** Scatter plots of correlations between areas at different levels of the upper airways on MRI and spirometry

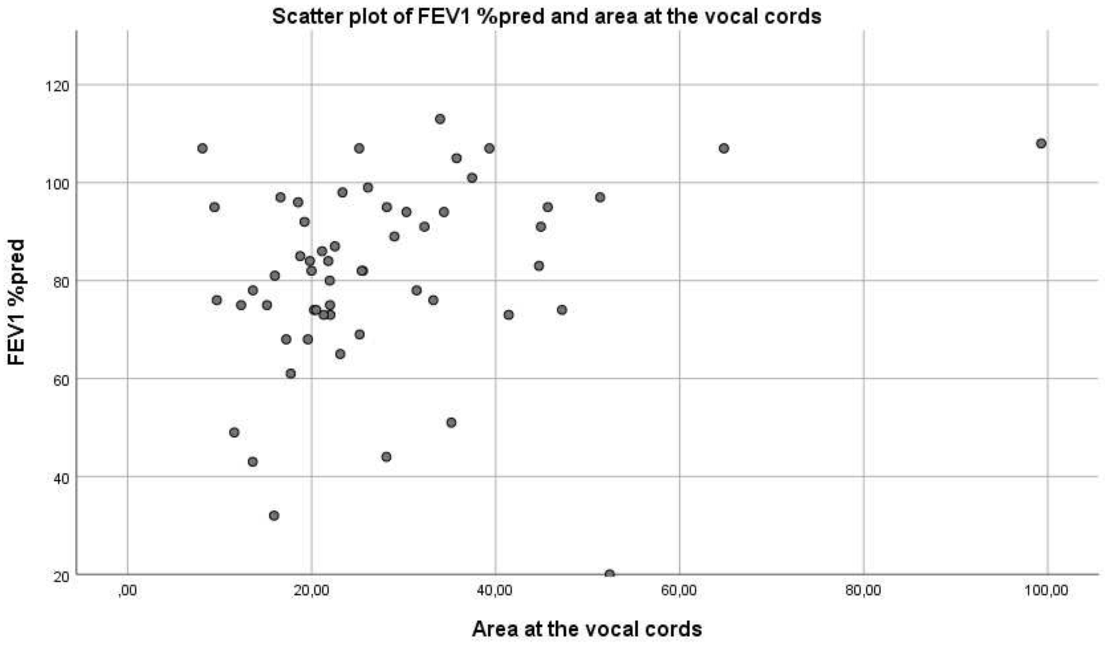


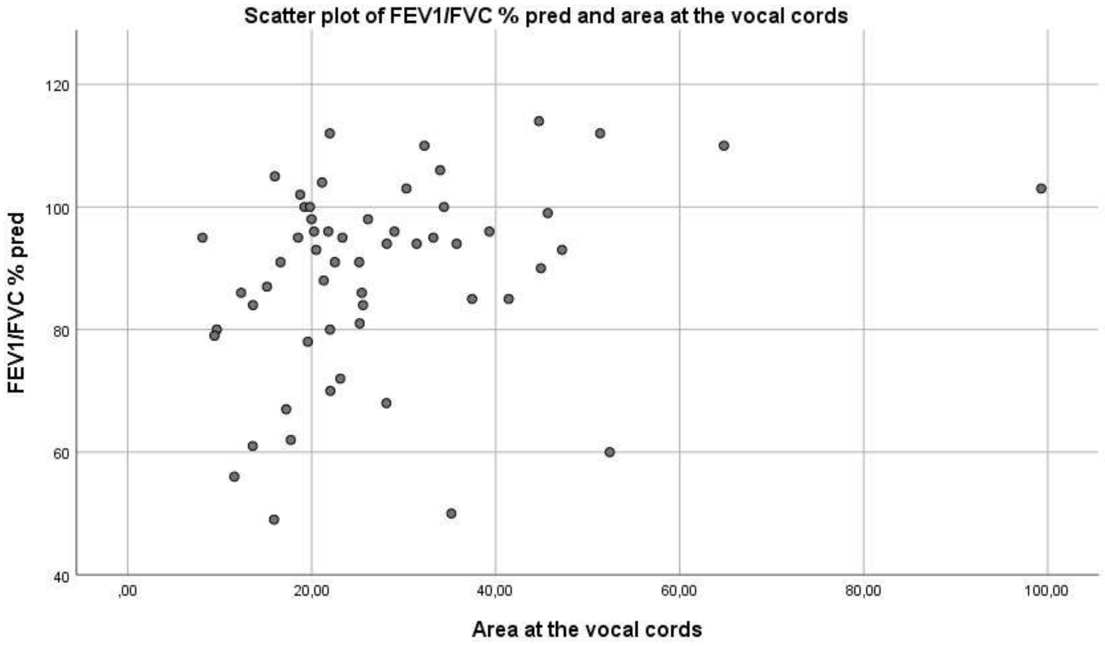

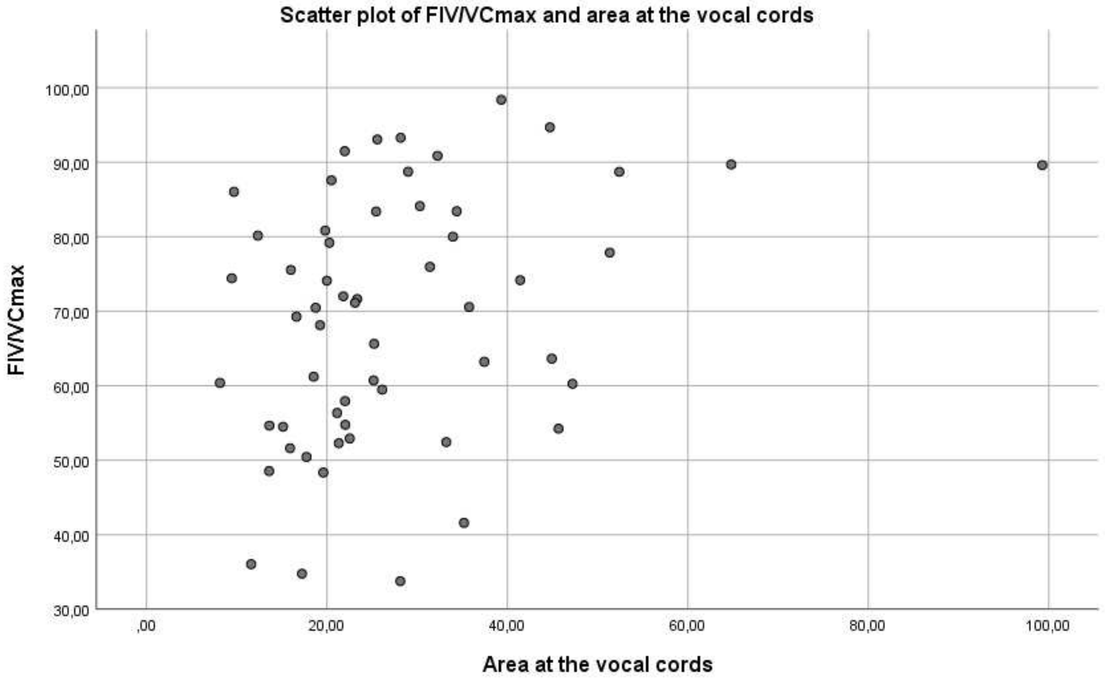
Scatter plot of FIV/VCmax and area at the vocal cords
FIV/VCmax
100,00
90,00
80,00
70,00
60,00
50,00
40,00
30,00
,00
20,00
40,00
60,00
80,00
100,00
Area at the vocal cords

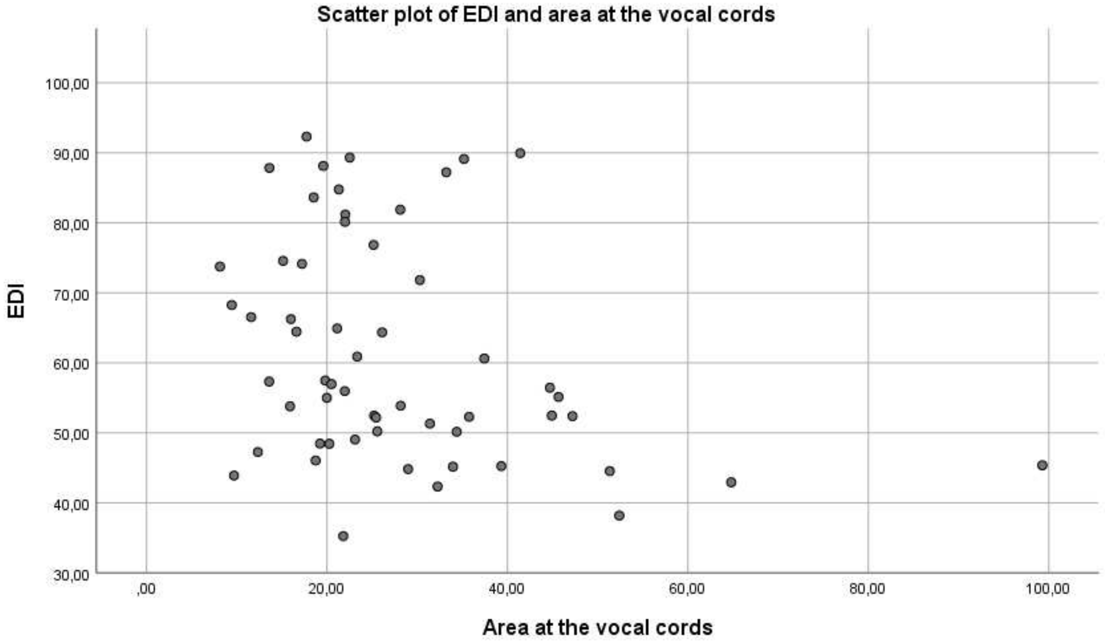
Scatter plot of EDI and area at the vocal cords
EDI
100,00
90,00
80,00
70,00
60,00
50,00
40,00
30,00
,00
20,00
40,00
60,00
80,00
100,00
Area at the vocal cords

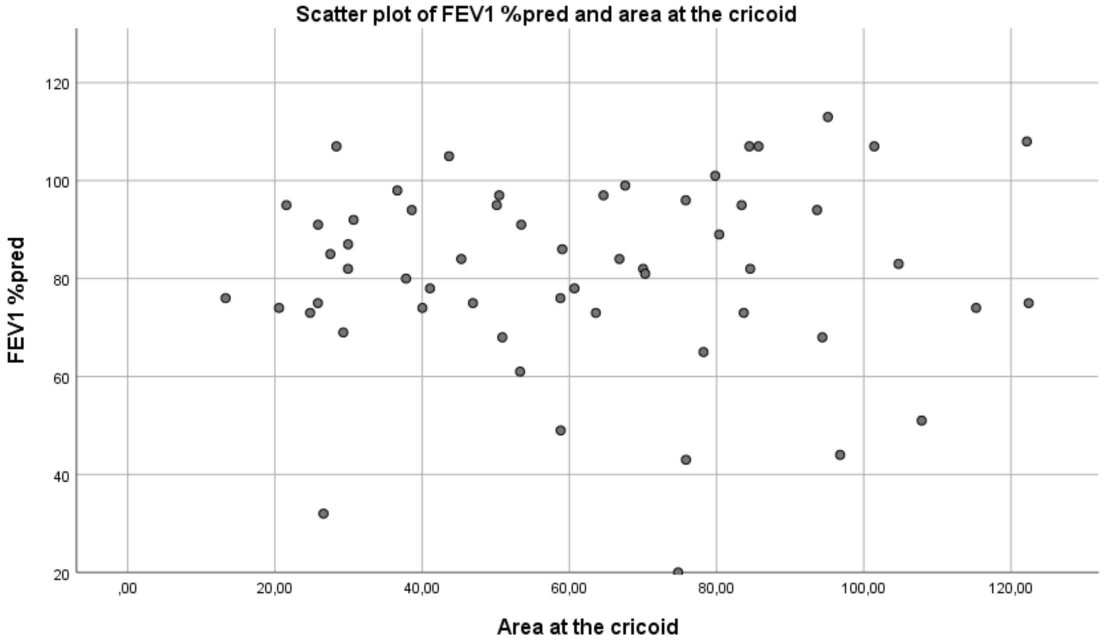
Scatter plot of FEV1 %pred and area at the cricoid
FEV1 %pred
120
100
80
60
40
20
,00
20,00
40,00
60,00
80,00
100,00
120,00
Area at the cricoid

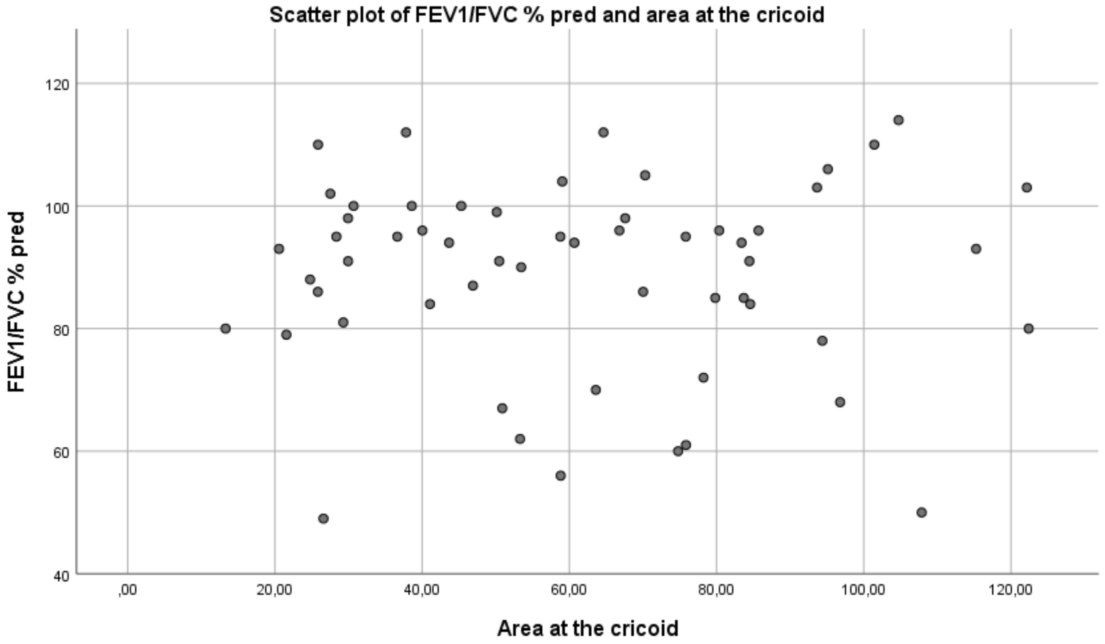
Scatter plot of FEV1/FVC % pred and area at the cricoid
FEV1/FVC % pred
120
100
80
60
40
,00
20,00
40,00
60,00
80,00
100,00
120,00
Area at the cricoid

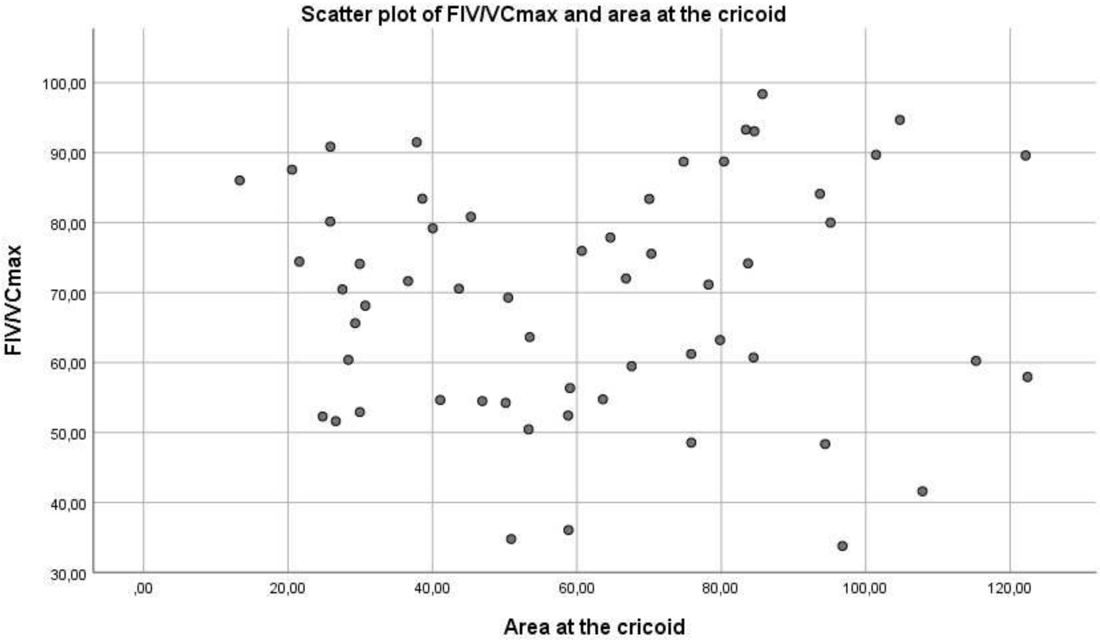
Scatter plot of FIV/VCmax and area at the cricoid
FIV/VCmax
100,00
90,00
80,00
70,00
60,00
50,00
40,00
30,00
,00
20,00
40,00
60,00
80,00
100,00
120,00
Area at the cricoid

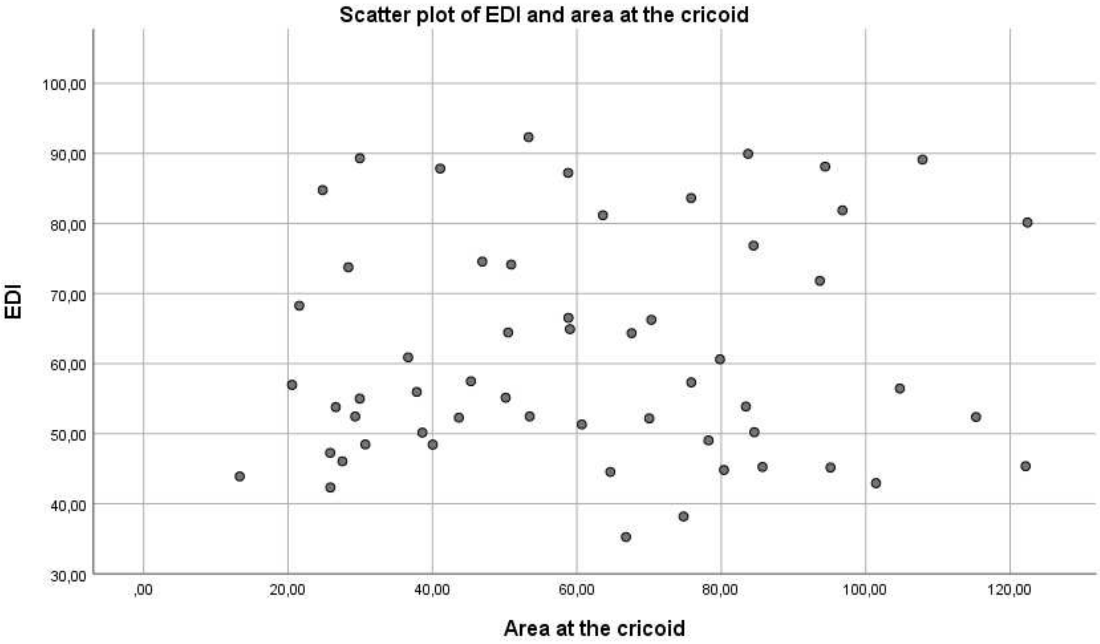
Scatter plot of EDI and area at the cricoid
EDI
100,00
90,00
80,00
70,00
60,00
50,00
40,00
30,00
,00
20,00
40,00
60,00
80,00
100,00
120,00
Area at the cricoid

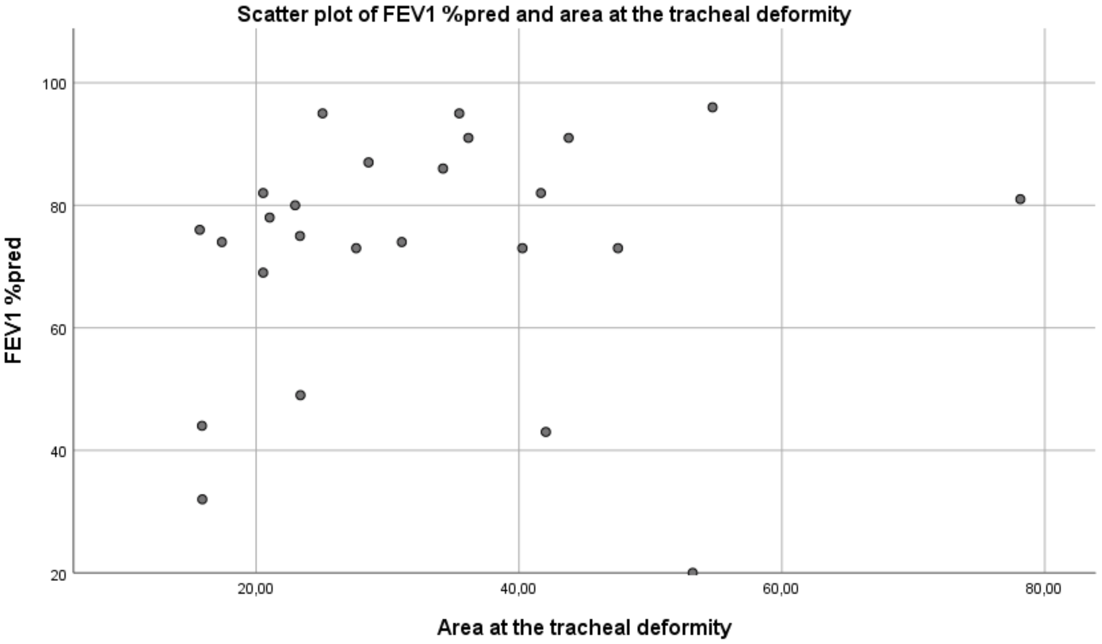
Scatter plot of FEV1 %pred and area at the tracheal deformity
FEV1 %pred
100
80
60
40
20
20,00
40,00
60,00
80,00
Area at the tracheal deformity

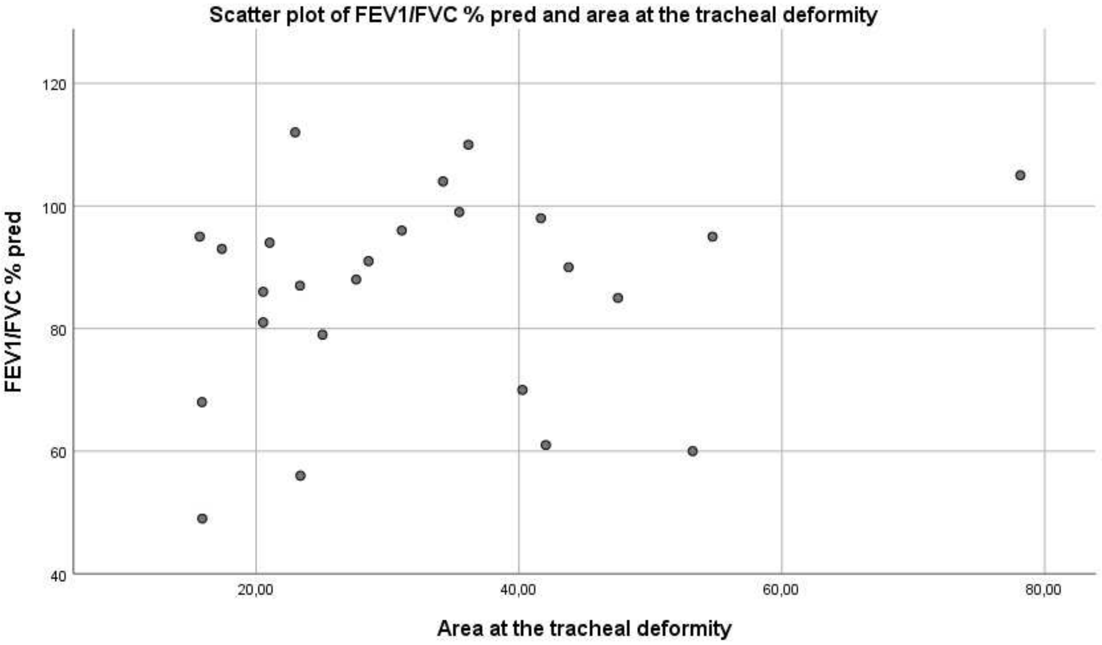
Scatter plot of FEV1/FVC % pred and area at the tracheal deformity
FEV1/FVC % pred
120
100
80
60
40
20,00
40,00
60,00
80,00
Area at the tracheal deformity

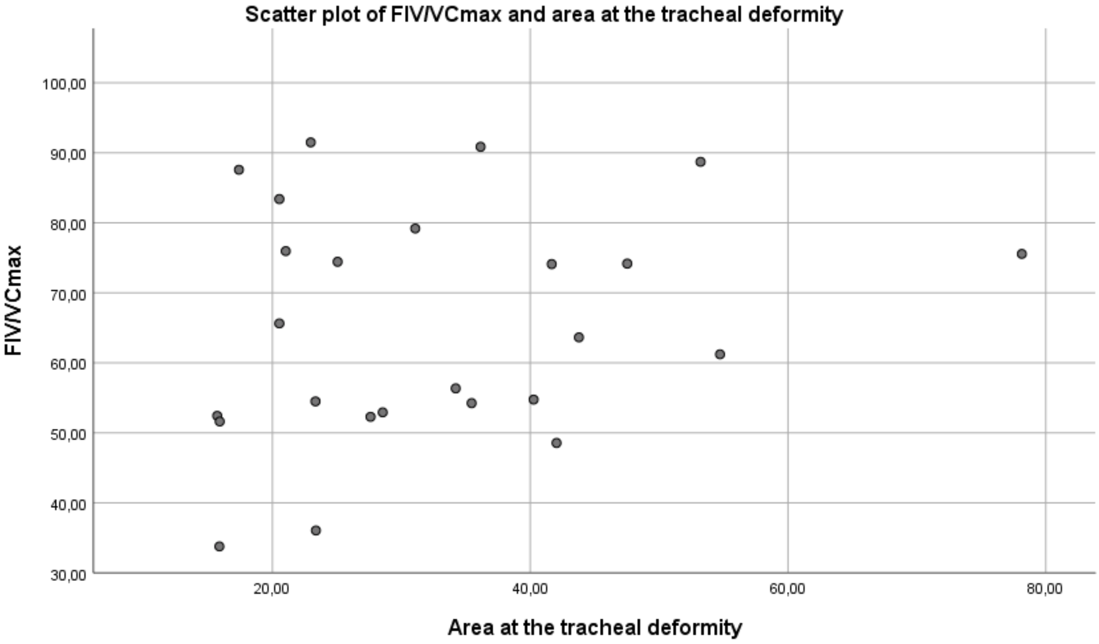
Scatter plot of FIV/VCmax and area at the tracheal deformity
FIV/VCmax
100,00
90,00
80,00
70,00
60,00
50,00
40,00
30,00
20,00
40,00
60,00
80,00
Area at the tracheal deformity

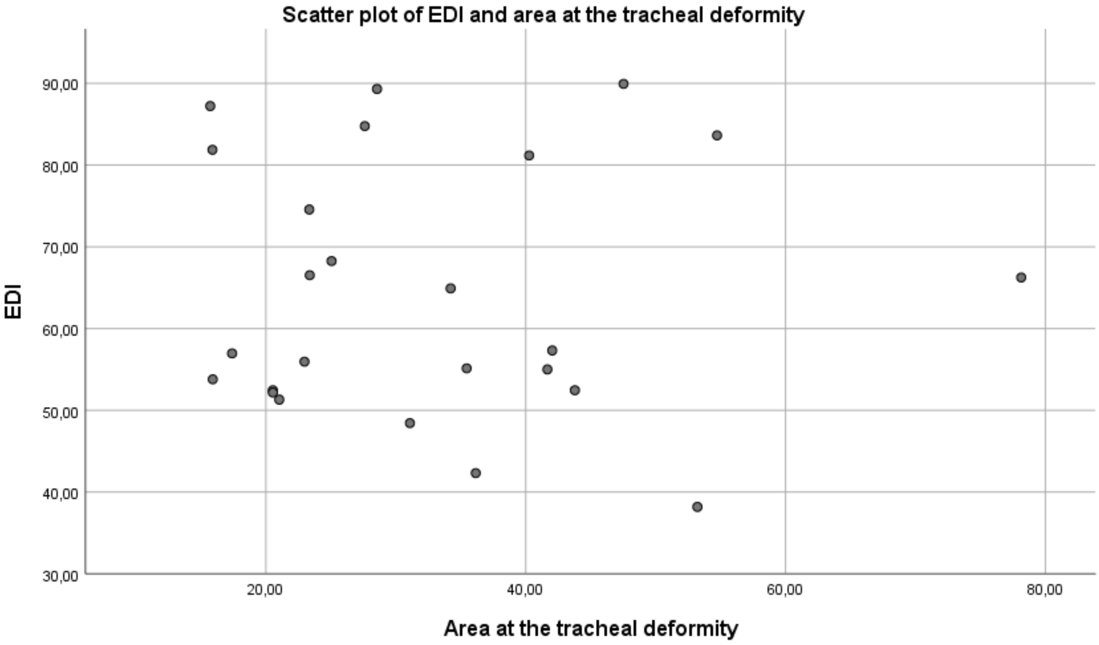
Scatter plot of EDI and area at the tracheal deformity
EDI
90,00
80,00
70,00
60,00
50,00
40,00
30,00
20,00
40,00
60,00
80,00
Area at the tracheal deformity

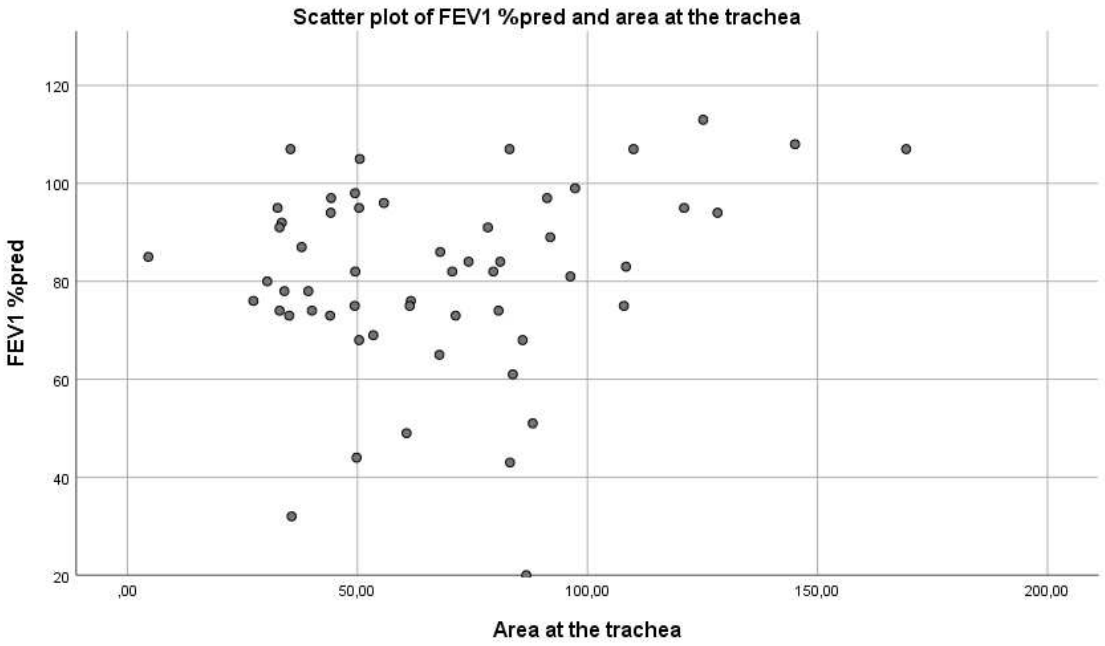
Scatter plot of FEV1 %pred and area at the trachea
FEV1 %pred
120
100
80
60
40
20
,00
50,00
100,00
150,00
200,00
Area at the trachea

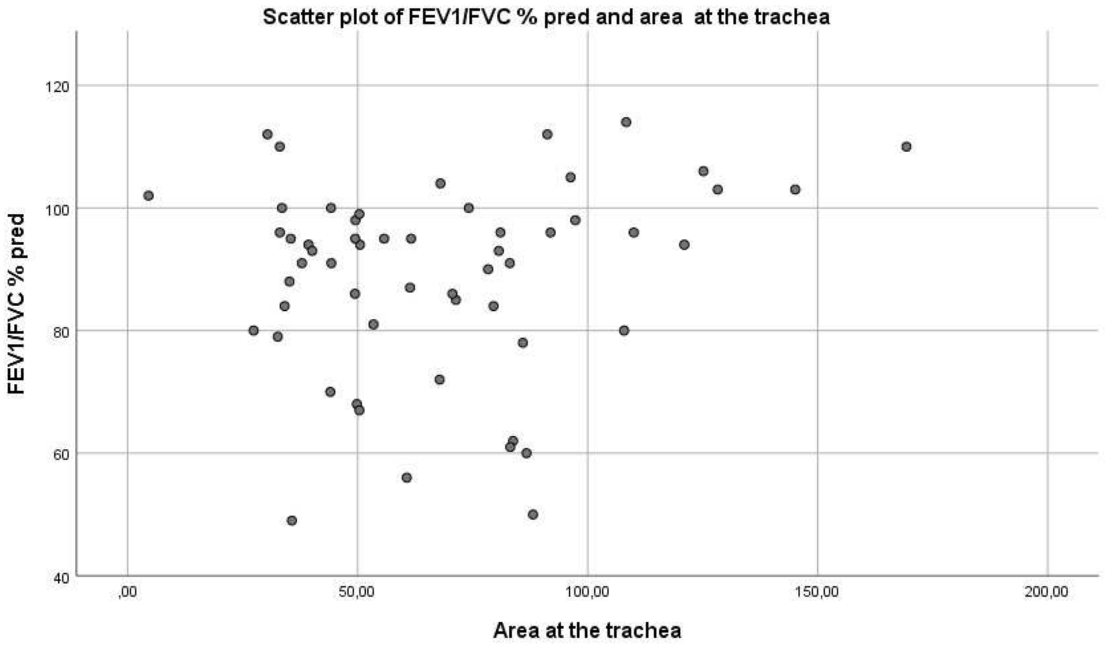
Scatter plot of FEV1/FVC % pred and area  at the trachea
FEV1/FVC % pred
120
100
80
60
40
,00
50,00
100,00
150,00
200,00
Area at the trachea

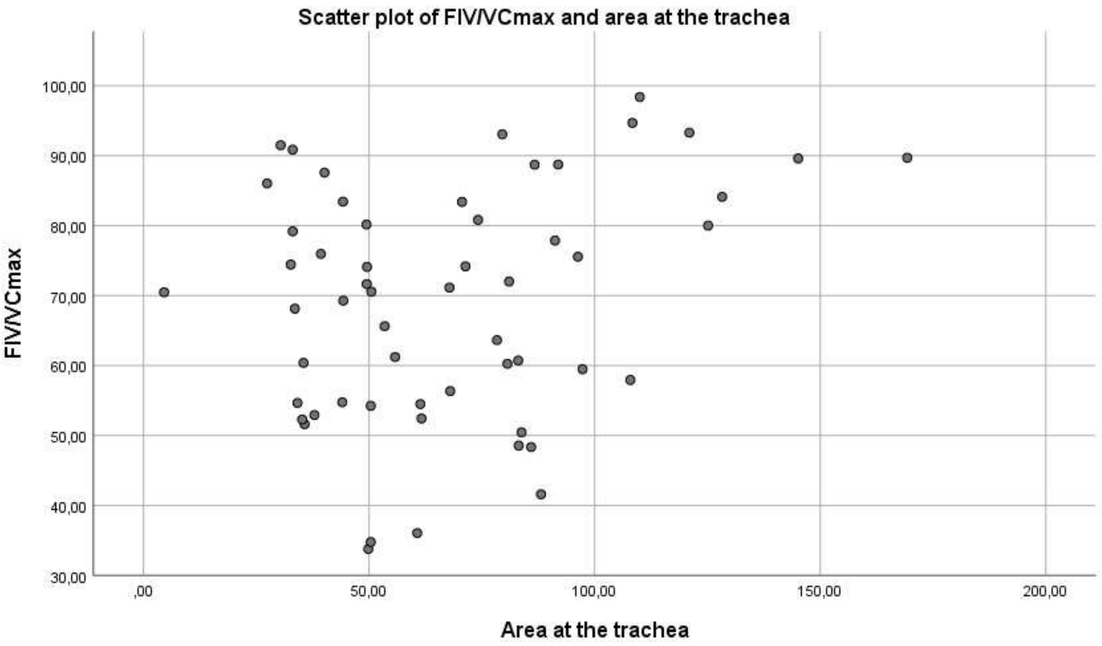
Scatter plot of FIV/VCmax and area at the trachea
FIV/VCmax
100,00
90,00
80,00
70,00
60,00
50,00
40,00
30,00
,00
50,00
100,00
150,00
200,00
Area at the trachea

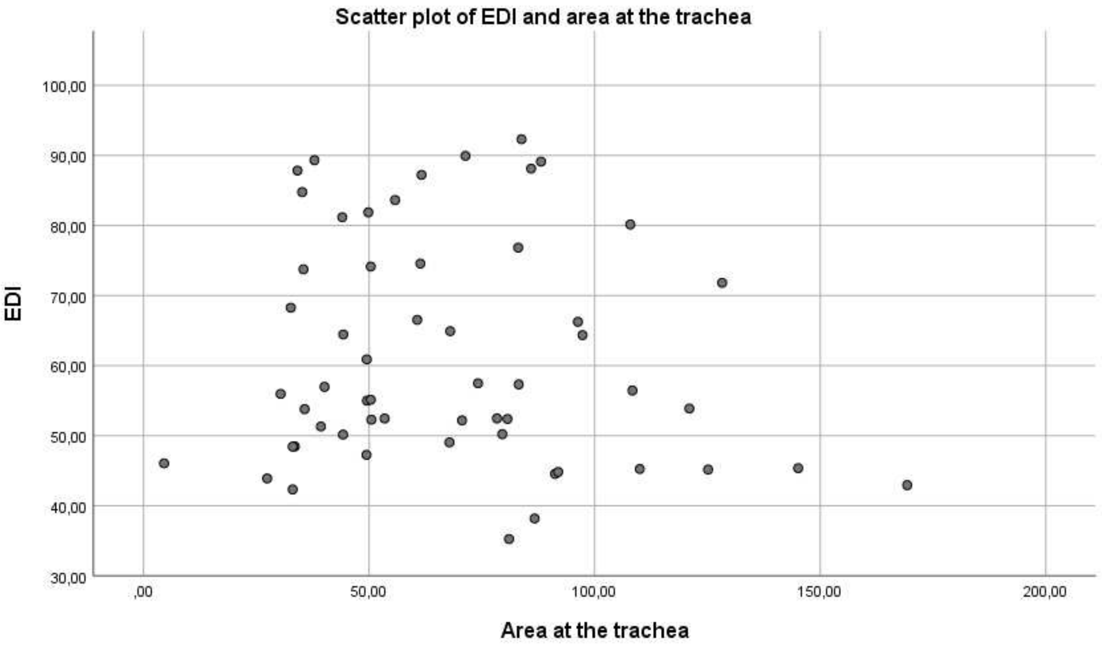
Scatter plot of EDI and area at the trachea
EDI
100,00
90,00
80,00
70,00
60,00
50,00
40,00
30,00
,00
50,00
100,00
150,00
200,00
Area at the trachea
