# Supplementary material for: MRI of the upper airways in children and young adults: the MUSIC study
Source: Thorax. 2020 Oct 29;76(1):44–52. doi: 10.1136/thoraxjnl-2020-214921 (PMC7803889; doi:10.1136/thoraxjnl-2020-214921)
Supplement: Supplementary data [file thoraxjnl-2020-214921supp007.pdf]

Online supplement 6: incidental finding of hypodense regions on proton density imaging

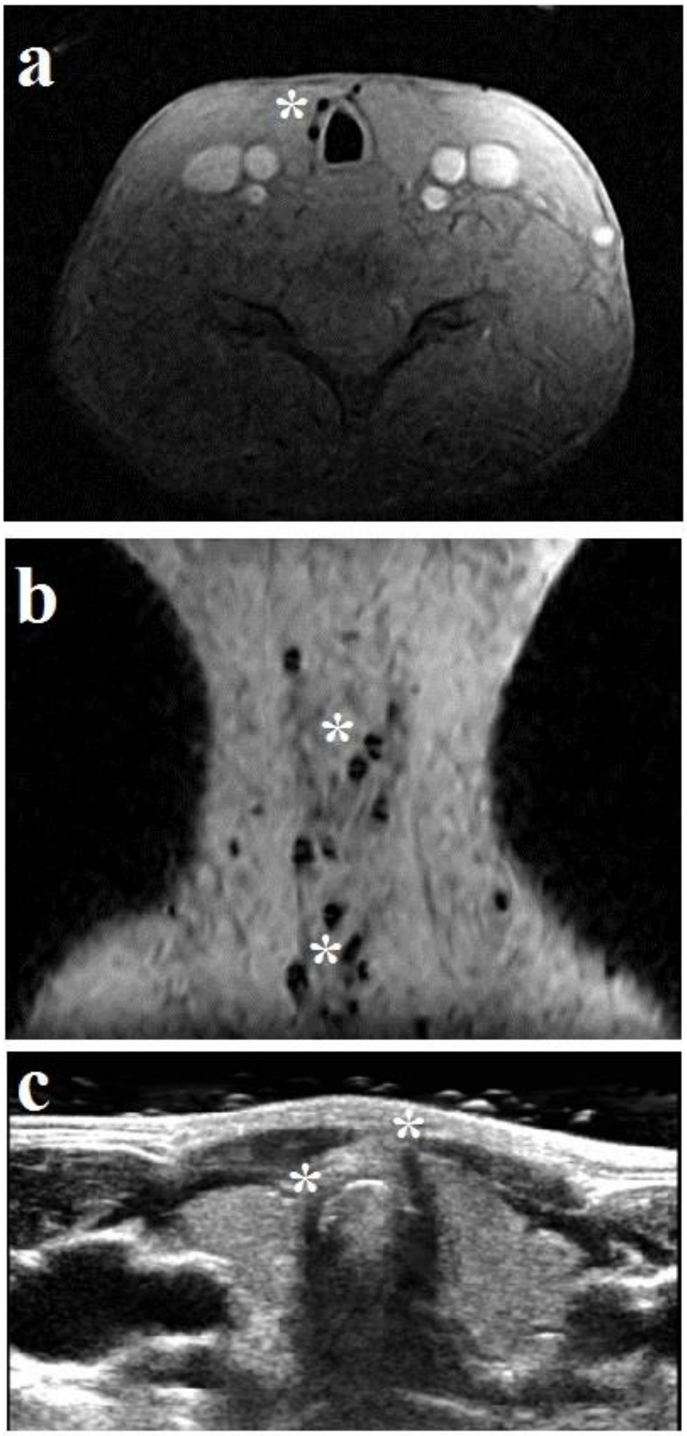

Online supplement 6: example of a patient with history of LTS showing an incidental finding of hypodense regions (*) on axial (A) and sagittal (B) proton density weighted MRI and corresponding ultrasonography (C).
