# Supplementary figures and images for: MRI of the upper airways in children and young adults: the MUSIC study
Source: Thorax. 2020 Oct 29;76(1):44–52. doi: 10.1136/thoraxjnl-2020-214921 (PMC7803889; doi:10.1136/thoraxjnl-2020-214921)

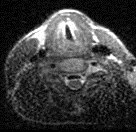

Supplement: Supplementary data [file thoraxjnl-2020-214921supp008.gif]
