# Supplementary material for: MRI of the upper airways in children and young adults: the MUSIC study
Source: Thorax. 2020 Oct 29;76(1):44–52. doi: 10.1136/thoraxjnl-2020-214921 (PMC7803889; doi:10.1136/thoraxjnl-2020-214921)
Supplement: Supplementary data [file thoraxjnl-2020-214921supp009.pdf]

Online supplement 7: Intra- and interclass variability

A: Intra and inter observer variability of static MRI measurements

	Intraclass correlation	Interclass correlation
**Presence of vocal cord thickening**	0.92	0.67
**Level of the vocal cords**		
Area	0.99	0.95
AP diameter	0.99	0.91
Transversal diameter	0.98	0.89
**Level of the cricoid**		
Area	0.92	0.97
AP diameter	0.97	0.97
Transversal diameter	0.92	0.93
**Presence of tracheal deformation**	1.00	1.00
**Level of tracheal deformation**	0.93	0.99
Area	0.61	0.99
AP diameter	0.89	0.99
Transversal diameter		
**Level of proximal trachea**		
Area	0.99	0.98
AP diameter	1.00	0.91
Transversal diameter	0.97	0.95

Data are presented as intra class correlation coefficient (ICC). AP; anterior- posterior.

B: Intra and inter observer variability of dynamic MRI measurements

	Intraclass correlation	Interclass correlation
**Complete abduction during inspiration**	0.42	0.65
**Complete adduction during phonation**	0.91	0.00
**Inspiration areas**		
Vocal cords	0.91	0.97
Cricoid	0.99	0.93
Tracheal deformation	-	-
Trachea	0.98	0.98
**Phonation areas**		
Vocal cords	0.92	0.94
Cricoid	0.95	0.99
Tracheal deformation	-	-
Trachea	1.00	0.99

Data are presented as intra class correlation coefficient (ICC).
